# Association of serum uric acid to high-density lipoprotein cholesterol ratio with all-cause mortality and cardiovascular disease mortality in patients with gout

**DOI:** 10.1186/s12872-025-05254-x

**Published:** 2025-11-19

**Authors:** Xiaolin Lou, Rubing Guo, Yongtong Cao, Wei Zhao

**Affiliations:** 1 School of Public Health, Gansu University of Traditional Chinese Medicine, Gansu 730000 Lanzhou, China; 2 Department of Clinical Laboratory, China-Japan Friendship Hospital, Beijing, 100029 China

**Keywords:** Gout, Serum uric acid, High-density lipoprotein cholesterol, All-cause mortality, Cardiovascular mortality

## Abstract

**Background:**

The uric acid to high-density lipoprotein cholesterol ratio (UHR), a novel biomarker, has clinical value in diagnosing and evaluating metabolic syndrome, diabetes mellitus, and atherosclerosis. However, the relationship between UHR and mortality in gout patients is unknown.This study aimed to investigate UHR’s link to all-cause and cardiovascular mortality in gout patients.

**Methods:**

Based on NHANES data from 2007 to 2018, 1,479 gout patients who completed follow-up as of December 31, 2018 were included, and patients were divided into four groups using UHR, we analyzed the relationship between UHR and risk of death using Cox regression models, tested for nonlinear relationships with a restricted cubic spline, and assessed for population heterogeneity by stratifying by sex, age, and body mass index.

**Results:**

The Kaplan-Meier analysis revealed that patients in the third UHR quartile (Q3) had the highest risk of death. The multivariate Cox regression analysis further confirmed that the overall mortality risk (HR: 1.44, 95% CI: 1.05–1.97) and cardiovascular mortality risk (HR: 1.73, 95% CI: 1.04–2.87) of this group were higher than those of the other groups. Restricted cubic spline analysis revealed a U-shaped association between UHR and all-cause mortality, with a significant positive correlation above a threshold of 21.6% (HR: 1.68, 95% CI: 1.26–2.24) and a linear positive correlation with cardiovascular mortality (HR: 1.40, 95% CI: 1.05–1.86). Subgroup analyses showed that these associations were consistent across age, BMI, and sex (*p* > 0.05 for interaction).

**Conclusion:**

This study confirms that UHR in gout patients has a U-shaped correlation with all-cause mortality and a linear correlation with cardiovascular mortality. Therefore, UHR may serve as a novel prognostic biomarker for mortality risk stratification in patients with gout.

**Supplementary Information:**

The online version contains supplementary material available at 10.1186/s12872-025-05254-x.

## Introduction

Gout is an inflammatory joint disease whose pathogenesis is closely related to the deposition of uric acid crystals in the joint cavity [1]. Increased concentrations of uric acid in the blood can promote the deposition of monosodium urate in the synovial fluid or soft tissues of the joints, thereby inducing a dramatic arthritic response [2, 3]. The prevalence of gout has been steadily climbing, contributing to a substantial disease burden globally [4]. It stands as the most prevalent inflammatory arthritis in men and ranks among the leading causes of arthritis in elderly women, particularly in high-income nations [5]. In the United States alone, an estimated 6.1 million adults grapple with this condition [4]. Emerging research suggests that systemic inflammation, mediated by endothelial dysfunction, may drive the development of atherosclerosis, reduce arterial flexibility, and impair blood flow, thereby accelerating the progression of cardiovascular disease. Accumulating evidence suggests that gout increases the risk of death [6]. Consequently, for individuals with uric acid metabolic dysfunction, early detection and timely intervention of related risk factors is the key to optimizing the therapeutic effect.

Recent studies have shown the value of UHR as a novel biomarker in assessing inflammatory response and metabolic status [7]. Uric acid (UA), a byproduct of purine metabolism, can contribute to atherosclerosis by oxidizing low-density lipoprotein cholesterol (LDL-C) and damaging vascular walls [8]. On the other hand, high-density lipoprotein cholesterol (HDL-c) offers cardioprotective benefits through its antioxidant and anti-inflammatory properties [9]. Lower HDL-c levels are significantly associated with a higher cardiovascular disease risk [10]. Nevertheless, the predictive accuracy of these markers is often compromised by comorbidities affecting kidney function and lipid metabolism [11–13]. This phenomenon strongly suggests the need to construct a more systematic risk evaluation system. UHR is a promising composite biomarker. Studies reveal that in patients with chronic kidney disease, UHR outperforms UA or HDL-c alone in predicting coronary artery disease [14]. In addition, it has been used in metabolic syndrome, nonalcoholic fatty liver disease, Hashimoto thyroiditis, and coronary collateral circulation [15–19]. Notably, peritoneal dialysis patients with high UHR are at a markedly increased risk of cardiovascular death [20].

As a straightforward and readily accessible indicator, UHR is increasingly recognized for its potential to predict and prognosticate a range of clinical outcomes. While earlier research has established that UHR can predict metabolic disorders and is linked to cardiovascular conditions [21, 22], its connection to both all-cause and cardiovascular mortality in gout patients remains underexplored. This study seeks to bridge that gap by investigating how UHR relates to these mortality outcomes in individuals suffering from gout.

## Materials and methods

### Data sources and study population

This investigation leveraged data from the National Health and Nutrition Examination Survey (NHANES), a comprehensive program designed to assess the health and nutritional well-being of adults and children across the United States. Conducted by the National Center for Health Statistics (NCHS), NHANES utilized a multi-stage stratified sampling technique to construct a representative data sample covering the entire United States. Information is gathered through a combination of in-home interviews and physical health examinations. Ethical oversight was provided by the NCHS Ethical Review Board, and all participants provided written consent prior to their involvement. The datasets used in this analysis, spanning from 2007 to 2018, are publicly accessible on the NHANES website (https://www.cdc.gov/nchs/nhanes/index.html).

**Fig. 1:**
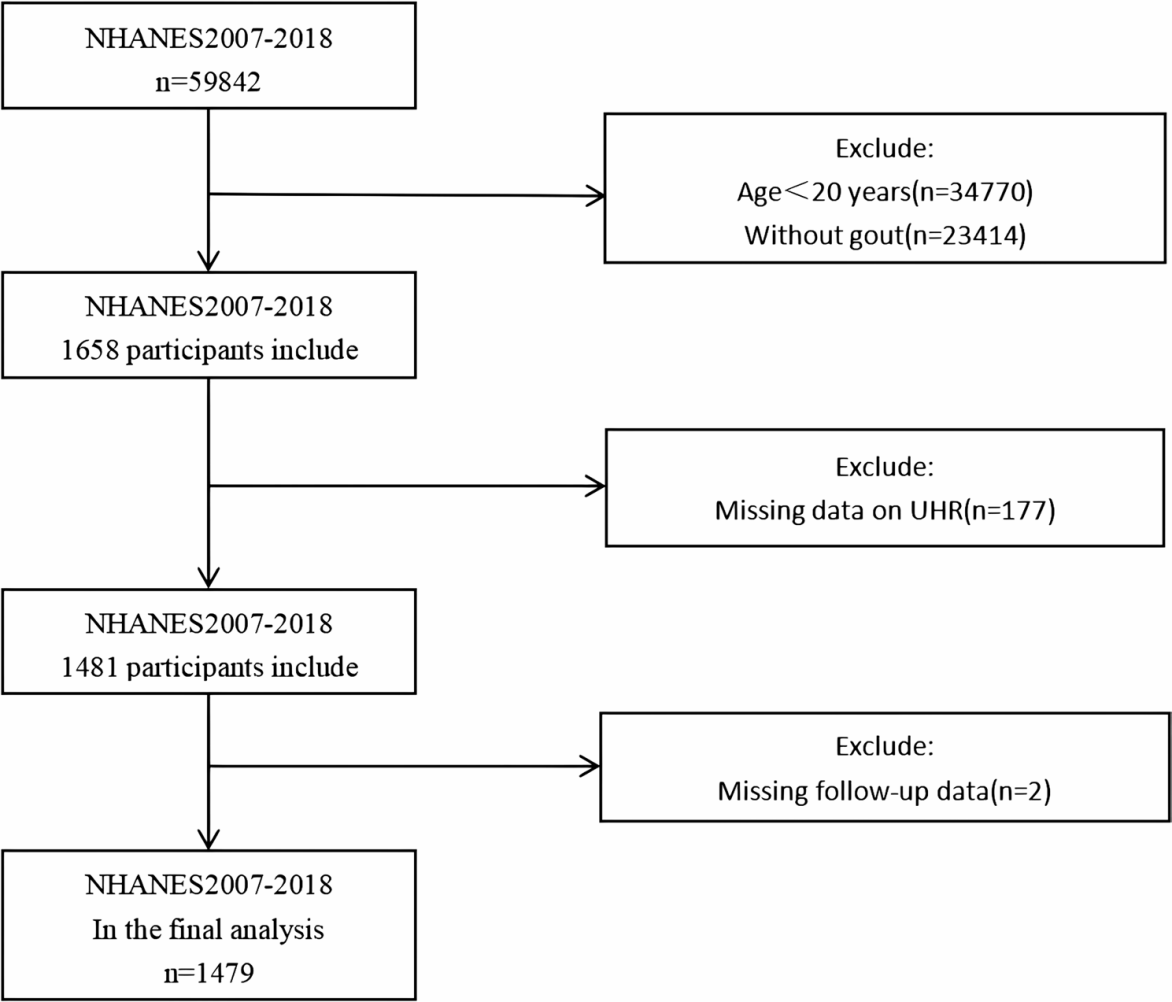
Flowchart of study participants selection

Gout diagnosis was ascertained using a self-reported questionnaire (MCQ160n), which posed the question: “Has a doctor or other health professional ever diagnosed you with gout?” Respondents were given a binary choice: “Yes” or “No.” Over the 12-year period analyzed, a total of 59,842 individuals were evaluated. Based on the diagnostic criteria, 1,658 adults aged 20 and older were initially identified as having gout. After excluding participants with missing UHR data (*n* = 177) and insufficient follow-up information (*n* = 2), the final sample comprised 1,479 individuals (Fig. 1). To address potential selection bias, we compared participants with and without UHR data (Table S2). While most baseline characteristics were comparable, excluded individuals had higher systolic blood pressure, a greater proportion of females and non-Hispanic Black participants, and a higher diabetes prevalence. Nonetheless, the overall similarities suggest that exclusion due to missing UHR likely did not substantially bias our main findings.

### UHR calculation

UHR is obtained by dividing UA by HDL-c. Serum UA concentration was quantified by time endpoint method, while HDL-c level was measured by direct immunoassay technique. For sample preservation details, assay procedures, and quality control, consult the relevant technical documents on the NHANES official website.

### Outcome assessment

The mortality data used in this study were obtained from the National Death Index (NDI) database maintained by the NCHS, Death cause codes follow the World Health Organization-issued International Classification of Diseases (ICD − 10) standards. Follow-up is from the date of baseline examination to the date of death or December 31, 2018, depending on which occurs first. Study outcomes encompassed all-cause mortality and cardiovascular mortality, with the latter including deaths attributed to heart diseases (ICD-10 codes I00-I09, I11, I13, I20-I51) and cerebrovascular diseases (ICD-10 codes I60-I69). Detailed statistics on mortality rates can be obtained by visiting the Death Information topic page on the CDC website (https://www.cdc.gov/nchs/data-linkage/mortality.htm).

### Covariates

The selection of covariates was based on the following twofold criterion: first, the corresponding regression coefficients of the variable changed by more than 10% when the variable was removed from the overall model, and second, the significance level of the regression coefficients of the variable needed to meet the statistical criterion of *p* < 0.1. These factors were deemed influential. Demographic variables included age, gender (male or female), and race (e.g., Mexican Americans, non-Hispanic whites, non-Hispanic blacks). Medical history was also recorded. Trained professionals measured participants’ height, weight, and blood pressure at mobile examination centers, with body mass index (BMI) calculated as weight (kg) divided by height squared (m²). Laboratory analyses covered a range of parameters: white blood cell count (WBC), red blood cell count (RBC), platelet count (PLT), neutrophil count (NE), serum sodium (Na), potassium (K), chloride (Cl), total protein (TP), UA, HDL-c, triglycerides (TG), alanine aminotransferase (ALT), lactate dehydrogenase (LDH), and creatinine (Scr). Comorbidities such as heart attacks, strokes, and cancers were also documented.

### Statistical analysis

Baseline characteristics were stratified based on UHR quartiles (3% ≤ Q1 < 10%, 10% ≤ Q2 < 14%, 14% ≤ Q3 < 19%, and 19% ≤ Q4 < 78%). Data were presented as means ± standard deviations for normally distributed variables, medians with interquartile ranges for skewed data, and frequencies with percentages for categorical variables. Normally distributed continuous variables were analyzed by analysis of variance (ANOVA), non-normally distributed data were tested by Kruskal-Wallis nonparametric test, and categorical variables were compared between groups by chi-square test. To ensure model stability, the covariates included in the model were diagnosed for multicollinearity using Variance Inflation Factor (VIF), and when the VIF value was greater than 10, the covariate was determined to have a significant multicollinearity problem and needed to be excluded from the model. We constructed three models: Model 1 was unadjusted, Model 2 adjusted for age and gender, and Model 3 further adjusted for smoking status, blood pressure metrics, race, hematological parameters, biochemical markers, and comorbidities such as cardiovascular disease and cancer. Based on restricted triple sampling to determine the relationship between UHR expression levels and cardiovascular and all-cause mortality. For possible nonlinear associations, segmented Cox regression analysis was applied to identify potential threshold effects. In addition, the results of survival analyses were visualized by Kaplan-Meier survival curves, and the log-rank test was used for statistical assessment of between-group differences. Study used age (60 vs. ≥ 60 years), gender (male vs. female) and BMI (28 kg/m² vs. ≥ 28 kg/m²) stratification to explore the relationship between UHR and mortality. To evaluate the stability of our results, we conducted several sensitivity analyses (Table S1), which included excluding extreme UHR values (Model 4), imputing missing UHR data using random forest (Model 5), and re-selecting covariates via LASSO regression (Model 6), with all models further adjusted for diabetes. Additionally, we compared baseline characteristics between participants with and without UHR data (Table S2) and computed incidence rates of mortality outcomes (per 1,000 person-years) by UHR quartiles (Table S3).Statistical significance was set at a two-sided *p*-value ≤ 0.05. All analyses were performed using R software version 4.3.2.

## Results

### Baseline characteristics of the study population

The research involved 1,479 participants, averaging 63.96 years old (± 12.98). The cohort was predominantly male, making up 69.64% of the group, while females represented 30.36%. UHR quartiles were categorized as follows: 3% ≤ Q1 < 10%, 10% ≤ Q2 < 14%, 14% ≤ Q3 < 19%, and 19% ≤ Q4 < 78%. The baseline characteristics are listed in Table 1. Participants in the highest quartile (Q4) showed significant differences in demographic characteristics: compared to the lowest quartile (Q1), subjects in the Q4 group tended to be younger in age structure, had a predominantly male gender distribution, and were predominantly non-Hispanic white. Additionally, individuals in Q4 exhibited higher levels of BMI, diastolic DBP, WBC, RBC, PLT, SCR, UA, HDL-c, ALT, TG, NE, alongside lower levels of LDH and SBP. Interestingly, the prevalence of comorbid conditions such as stroke (dropping from 14.91% to 8.89%) and cancer (falling from 22.43% to 14.86%) showed a marked decline. With the exception of serum sodium, serum potassium, chloride, and a history of heart disease, all other variables demonstrated statistically significant differences across the four groups (*p* < 0.05).

**Table 1:** Characteristics of participants according to UHR quartiles

Characteristic	UHR (%) quartiles				*P*-value
	Q1	Q2	Q3	Q4	
N of participants	370	369	369	371	
Age (years)	65.68 ± 12.52	65.65 ± 11.41	64.20 ± 12.68	60.32 ± 14.44	< 0.001
SBP (mmHg)	132.9 ± 22.19	135.3 ± 21.28	133.9 ± 19.80	129.7 ± 20.38	0.003
DBP (mmHg)	68.46 ± 16.00	70.35 ± 14.37	70.61 ± 14.72	71.11 ± 15.46	0.104
WBC count (10^9^/l)	7.31 ± 2.24	7.08 ± 2.08	7.57 ± 2.70	7.70 (6.30–9.10)	< 0.001
RBC count (10^12^/l)	4.46 ± 0.54	4.54 ± 0.54	4.67 ± 0.55	4.75 ± 0.65	< 0.001
Platelet count (10^9^/l)	232.9 ± 73.30	221.4 ± 61.40	221.9 ± 60.64	235.4 ± 72.34	0.005
Cre (mg/dL)	0.93 (0.77–1.15)	1.01 (0.84–1.23)	1.07 (0.90–1.28)	1.12 (0.96–1.37)	< 0.001
ALT (U/L)	19.00 (15.00–25.00)	22.00 (16.00–29.00)	22.00 (17.00–30.00)	24.00 (18.00–31.00)	< 0.001
TG (mg/dL)	109.00 (74.25–157.00)	138.00 (98.00–200.00.00.00)	159.00 (114.00–239.00.00.00)	201.00 (144.50–304.00)	< 0.001
LDH (U/L)	148.27 ± 40.84	146.17 ± 36.52	142.46 ± 35.47	140.32 ± 41.51	0.025
Na + (mmol/L)	139.40 ± 3.16	139.69 ± 2.47	139.57 ± 2.66	139.29 ± 2.68	0.203
K + (mmol/L)	4.08 ± 0.43	4.10 ± 0.43	4.07 ± 0.40	4.12 ± 0.41	0.428
Cl^−^ (mmol/L)	102.43 ± 3.86	103.05 ± 3.54	102.74 ± 3.58	102.75 ± 3.70	0.155
TP (g/L)	7.03 ± 0.52	7.13 ± 0.50	7.11 ± 0.49	7.26 ± 0.57	< 0.001
Neutrophils count (10^9^/l)	4.49 ± 1.91	4.20 ± 1.57	4.52 ± 1.74	4.81 ± 1.78	< 0.001
BMI (kg/m^2^)	29.30 ± 7.53	31.14 ± 7.16	32.63 ± 6.43	33.78 ± 7.74	< 0.001
HDL-c (mg/dL)	64.56 ± 16.56	50.69 ± 10.84	44.27 ± 8.24	35.27 ± 6.87	< 0.001
UA (mg/dL)	4.81 ± 1.32	6.0 ± 1.28	7.10 ± 1.26	8.35 ± 1.49	< 0.001
Sex (%)					< 0.001
Male	51.89%	64.77%	76.69%	85.18%	
Female	48.11%	35.23%	23.31%	14.82%	
Race (%)					0.125
Mexica American	5.41%	5.96%	8.13%	9.16%	
Other Hispanic	4.86%	7.59%	5.69%	5.66%	
Non-Hispanic White	49.73%	47.43%	50.14%	50.67%	
Non-Hispanic Black	30.27%	27.64%	23.04%	21.29%	
Others	9.73%	11.38%	13.01%	13.21%	
Heart attack (%)					0.913
Yes	14.67%	14.99%	14.40%	16.17%	
No	85.33%	85.01%	85.60%	83.83%	
Stroke (%)					0.034
Yes	14.91%	11.92%	9.24%	8.89%	
No	85.09%	88.08%	90.76%	91.11%	
Cancer (%)					0.008
Yes	22.43%	18.70%	24.12%	14.86%	
No	77.57%	81.30%	75.88%	85.14%	
Diabetes					
Yes	27.84%	31.71%	36.04%	32.35%	0.124
No	72.16%	68.29%	63.96%	67.65%	

Continuous variables with a normal distribution were reported as mean ± standard deviation (SD), whereas skewed continuous variables were presented as medians (interquartile range [IQR]). Categorical data are presented as n (%). To assess the presence of statistically significant differences among the tertiles, the c2-test was employed for categorical variables, the Kruskal–Wallis test was used for skewed variables, and one-way analysis of variance (ANOVA) was applied for variables with a normal distribution

*UA* Uric acid, *HDL-c* high-density lipoprotein cholesterol, *DBP* diastolic blood pressure, *SBP* systolic blood pressure, *RBC count* Red blood cell count, *WBC count* White blood cell count, *Cre* Creatinine, *TG* Triglycerides, *LDH* Lactate dehydrogenase, *Na*^+^ Sodium, *K*^+^ Potassium, *Cl*^-^ Chloride, *TP* Total protein

### Relationship between UHR and mortality

**Fig. 2:**
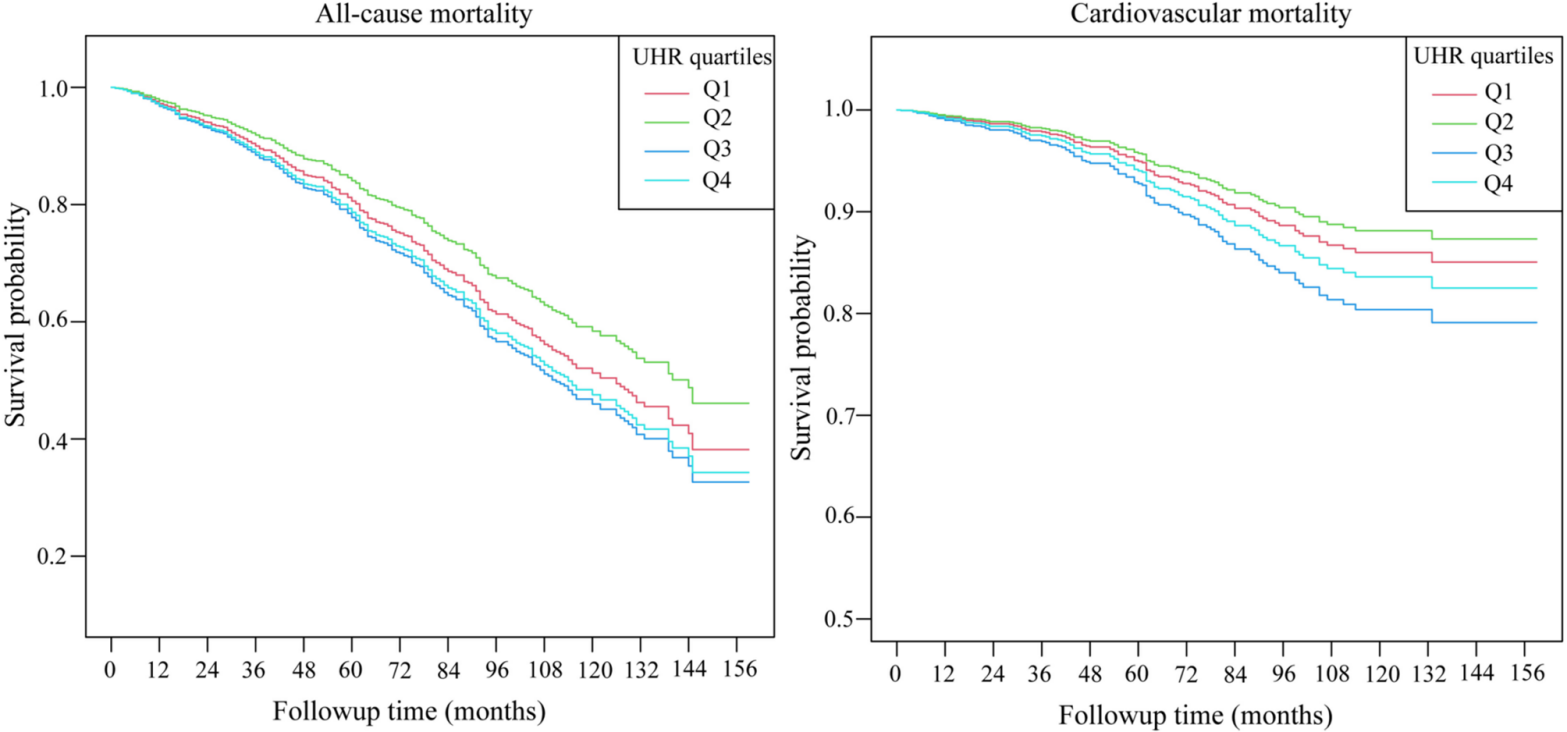
Kaplan–Meier survival curve for all-cause and cardiovascular mortality by UHR quartiles

During a mean follow-up of 70.45 months, 358 deaths occurred, including 127 cardiovascular deaths. Kaplan-Meier analysis (Fig. 2) showed that individual survival was significantly decreased in the third quartile of UHR (Q3) (*P* = 0.022). In this study, three Cox proportional risk regression models were constructed using the second quartile (Q2) as the reference group to delve into the correlation between the UHR metrics and the risk of death. See Table 2 for details. In the initial uncorrected model, UHR metrics were not significantly associated with the risk of both all-cause mortality and cardiovascular disease mortality (*P* > 0.05). However, in the fully corrected model 3, UHR levels were positively associated with all-cause mortality (HR: 1.26, 95% CI: 1.06–1.50, *P* = 0.009) and cardiovascular mortality (HR: 1.40, 95% CI: 1.05–1.86, *P* = 0.020). Sensitivity analyses using UHR as a categorical variable further validated this result. In fully adjusted model 3, compared with the second quartile (Q2), the study population in the third quartile (Q3) had a 44% increased risk of all-cause mortality (HR: 1.44, 95% CI: 1.05–1.97, *P* = 0.022) and a 73% increased risk of cardiovascular disease-related mortality (HR: 1.73, 95% CI: 1.04–2.87, *P* = 0.035).

**Table 2:** Association between UHR and risks of mortality in patients with gout

UHR (10%) quantiles	Model1		Model2		Model3	
	HR (95%CI)	*P*-value	HR (95%CI)	*P*-value	HR (95%CI)	*P*-value
All-cause mortality						
UHR (per 10% increment)	0.97 (0.83, 1.13)	0.714	1.22 (1.03, 1.43)	0.018	1.26 (1.06, 1.50)	0.009
UHR quartile						
Q1	1.37 (1.02,1.84)	0.034	1.38 (1.02,1.85)	0.034	1.24 (0.90,1.70)	0.181
Q2	Reference		Reference		Reference	
Q3	1.15 (0.85,1.54)	0.370	1.25 (0.93,1.69)	0.139	1.44 (1.05,1.97)	0.022
Q4	1.02 (0.76,1.37)	0.900	1.35 (1.00,1.82)	0.047	1.38 (1.00,1.91)	0.053
P for trend		0.802		0.076		0.028
Cardiovascular mortality						
UHR (per 10% increment)	1.00 (0.78, 1.29)	0.972	1.27 (0.97, 1.65)	0.082	1.40 (1.05, 1.86)	0.020
UHR quartile						
Q1	1.15 (0.70,1.90)	0.575	1.13 (0.69,1.87)	0.627	1.19 (0.71,2.02)	0.511
Q2	Reference		Reference		Reference	
Q3	1.22 (0.76,1.97)	0.411	1.33 (0.82,2.15)	0.241	1.73 (1.04,2.87)	0.035
Q4	0.92 (0.56,1.52)	0.748	1.23 (0.75,2.04)	0.413	1.42 (0.81,2.47)	0.220
P for trend		0.831		0.310		0.092

Significance differences were shown as *P* < 0.05 marked in bold

*HR* hazard ratio, *95% CI* 95% confidence interval, *UHR* uric acid to high-density lipoprotein cholesterol ratio

Model 1: adjusted for none

Model 2: adjusted for age and gender

Model 3: adjusted for age, gender, race, DBP, SBP, WBC count, RBC count, Platelet count, Neutrophils count, Cre, ALT, LDH, TG, K^+^, Cl^-^, Na^+^, TP, heart attack, stroke, cancer

### Sensitivity analysis

To verify the robustness of the results, we conducted multiple sensitivity analyses (Supplementary Table S1). All sensitivity analysis models (Model 4, 5, and 6) further adjusted for diabetes status on the basis of the basic model to reduce potential confounding bias. First (Model 4), after excluding three patients with extreme UHR values, the main conclusion remained unchanged. Second (Model 5), after reanalyzing the data using random forest imputation to handle the missing UHR data (*n* = 177), the results still showed a significant positive correlation between UHR and all-cause mortality (HR: 1.25, 95% CI: 1.05–1.48; *p* = 0.010), and the association between UHR and cardiovascular disease mortality remained significant as well (for every 10% increase, HR: 1.44, 95% CI: 1.10–1.90; *p* = 0.008). Finally (Model 6), after reselecting covariates using LASSO regression, UHR was still significantly positively correlated with all-cause mortality (for every 10% increase, HR: 1.19, 95% CI: 1.01–1.40; *p* = 0.042). These results collectively indicate that our main findings are robust after controlling for multiple confounding factors, including diabetes.In addition, the all-cause mortality and cardiovascular disease mortality rates, as well as their incidence densities (per 1000 person-years), for each of the different UHR groups are presented in Supplementary Table S3. There were no significant differences in the crude incidence rates among the four groups.

### Threshold effect analysis

**Fig. 3:**
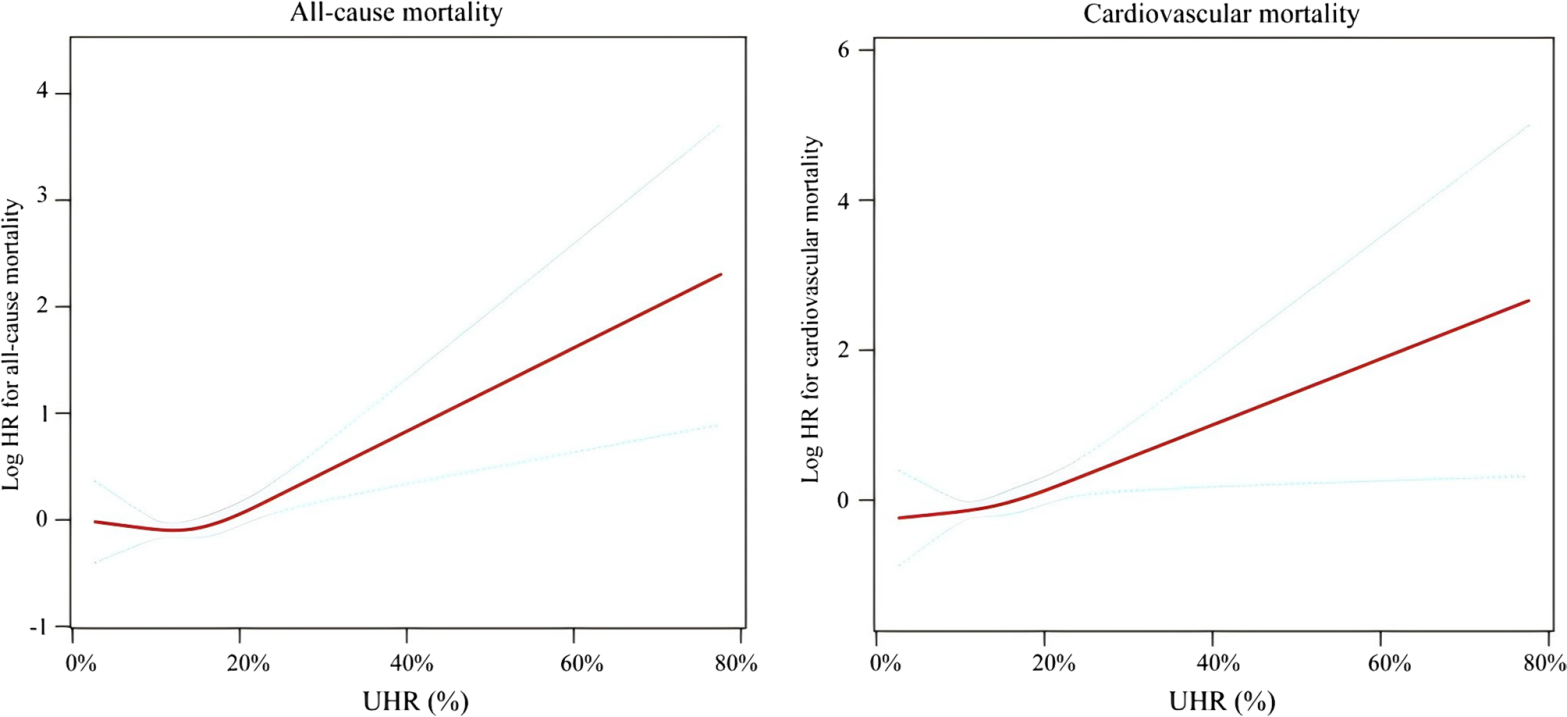
Dose-response relationship between serum UHR (%) and all-cause and CVD mortality in participants with gout. The solid and dotted lines represent the estimated values and their corresponding 95% CIs, respectively

A cubic spline model revealed a U-shaped relationship between UHR and all-cause mortality in gout patients (Fig. 3). Two-stage Cox proportional risk regression analysis (Table 3) identified a critical threshold of 21.6% (log-likelihood ratio *P* = 0.009). There was a corresponding 68% increase in the risk of all-cause mortality for each 10% increase in UHR above this threshold (HR: 1.68, 95% CI: 1.26–2.24, *P* < 0.001), whereas there was no significant association below this threshold (*P* < 0.05). In addition, there was a sustained positive association between UHR and the risk of cardiovascular death in gout patients (Fig. 3). For every 10% increase in UHR, the risk of cardiovascular death increased by 40%. (HR: 1.40, 95% CI: 1.05–1.86, *P* = 0.020).

**Table 3:** Threshold effect analysis of UHR on all-cause and cardiovascular mortality in patients with gout

	HR (95%CI)	*P*-value
All-cause mortality (per 10% increment)		
Fitting by the standard Cox proportional risk model	1.26 (1.06,1.50)	0.009
Fitting by the two-piecewise Cox proportional risk model		
Inflection point	21.6%	
UHR < 21.6%	1.05 (0.82,1.34)	0.709
UHR ≥ 21.6%	1.68 (1.26,2.24)	< 0.001
P for Log-likelihood ratio	0.041	
Cardiovascular mortality (per 10% increment)		
Fitting by the standard Cox proportional risk model	1.40 (1.05,1.86)	0.020
Fitting by the two-piecewise Cox proportional risk model		
Inflection point	22%	
UHR < 22%	1.12 (0.75,1.66)	0.585
UHR ≥ 22%	1.95 (1.25,3.04)	0.003
P for Log-likelihood ratio	0.118	

Adjusted for age, gender, race, DBP, SBP, WBC count, RBC count, Platelet count, Neutrophils count, S, ALT, LDH, TG, K^+^, Cl^-^, Na^+^, TP, heart attack, stroke, cancer

Significance differences were shown as *P* < 0.05 marked in bold

*HR* hazard ratio, *95% CI* 95% confidence interval, *UHR* uric acid to high-density lipoprotein cholesterol ratio

### Subgroup analysis

**Fig. 4:**
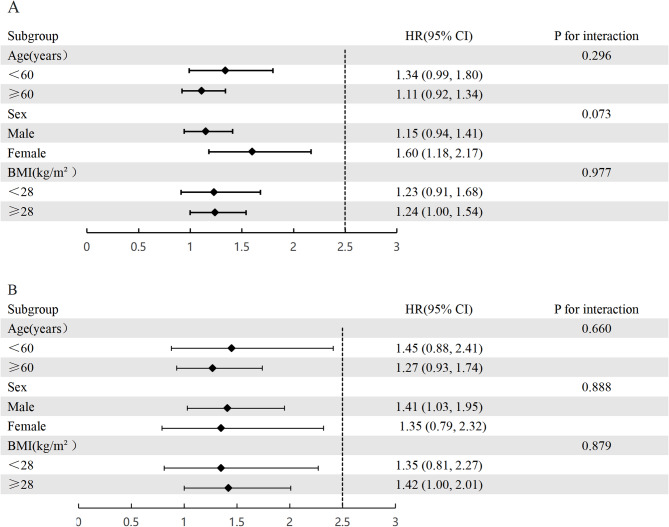
Subgroup analysis of UHR and all-cause mortality (**A**) and cardiovascular mortality (**B**)

These subgroups were categorized based on distinct attributes (Fig. 4): age (under 60 years old, 60 years and older), sex (male, female), and BMI (below 28 kg/m², 28 kg/m² and above). Interaction tests revealed that the association between UHR levels and mortality outcomes remained steady across all subgroups, showing no significant variation influenced by age, sex, or BMI (P for interaction > 0.05). These results show that the associations between UHR levels and all-cause and cardiovascular deaths in patients with gout are extremely robust and are applicable in patient populations from various types of clinical backgrounds.

## Discussion

Our study found that individuals with a baseline UHR between 14% and 19% had a significantly lower probability of survival. UHR showed a nonlinear U-shaped relationship with all-cause mortality (inflection point of 21.6%); it was linearly and positively associated with cardiovascular mortality. This study suggests that UHR is a strong predictor of all-cause and cardiovascular mortality in gout patients.

Research has consistently shown that people with high UA and low HDL-c levels face greater risks of both all-cause and cardiovascular-related deaths [23]. In a comprehensive study conducted in northern China, Li et al. uncovered a U-shaped link between HDL-c levels and all-cause mortality, particularly among younger adults [24]. Echoing these findings, Bowe et al. observed a comparable U-shaped correlation linking HDL-c to all-cause mortality across diverse kidney function stages [25]. Meanwhile, an Italian prospective study highlighted UA as a standalone predictor of all-cause mortality in diabetic patients [26]. Song et al. further reinforced this by establishing strong ties between UA levels and mortality risks in individuals with cardiovascular disease (CVD) [27]. However, the relationship becomes more nuanced in those with impaired uric acid metabolism. A large-scale study on gout patients found that those with UA levels under 0.36 mmol/L faced significantly higher risks of overall and cardiovascular mortality [28], possibly due to endothelial dysfunction caused by low uric acid [29]. These conflicting results imply that relying solely on UA or HDL-c may not be the most effective way to gauge mortality risks in gout patients. Instead, combining these two markers—specifically through the UHR—could offer a more accurate prognostic tool. UHR, a recently introduced inflammatory and metabolic indicator, has been linked to issues like poor glycemic control, metabolic syndrome, ischemic heart disease, and hypertension management [19–31]. Previous studies have highlighted UHR’s reliability in predicting all-cause mortality and cardiovascular risks across various patient groups. Elevated UHR levels significantly increase the probability of adverse cardiovascular outcomes and death in patients with moderate-to-severe chronic kidney disease (CKD) [32] those undergoing peritoneal dialysis [33], and individuals with chronic total occlusion (CTO) lesions [15]. The observed U-shaped relationship between UHR and all-cause mortality warrants further mechanistic interpretation.The low UHR end (< 21.6%) indicates low uric acid levels, which may lead to weakened endogenous antioxidant defense capacity [34]; the high UHR end (≥ 21.6%) represents the coexistence of high SUA and low HDL-c: SUA has pro-inflammatory and pro-oxidative properties [35], while low HDL-c weakens its cardiovascular protective effect [36], and the two together promote atherosclerosis and an increase in the risk of death. Our research further supports UHR’s role as a critical predictor of mortality and cardiovascular outcomes in diverse patient populations, solidifying its value as a key prognostic marker. This study pioneered a systematic analysis of the correlation between UHR indicators and survival prognosis of gout patients, filling a research gap in this field.

Our research, which tracked 1,479 U.S. adults with gout over an average follow-up period of approximately 70.45 months, found that higher UHR levels were correlated with elevated risks of both all-cause and cardiovascular mortality. The prognostic value of UHR was initially explored in the previous literature. Ding et al. [37] found a U-shaped association between UHR and all-cause and cardiovascular mortality in diabetic patients (inflection point values: 12.57%, 9.86%). The present study further revealed that UHR in individuals with gout exhibited a U-shaped correlation with overall mortality, with a tipping point at 21.6%, while demonstrating a straightforward positive link with deaths related to cardiovascular issues. Interestingly, the inflection point for all-cause mortality risk in gout patients was significantly higher than that in diabetics (21.6% vs. 12.57%), with fundamentally different cardiovascular risk patterns. This divergence likely arises from the unique pathophysiological mechanisms of each condition. Gout primarily involves inflammatory responses triggered by urate crystal deposits in joints and surrounding tissues [38, 39], whereas diabetes is characterized by chronic glucose metabolism disruptions leading to vascular endothelial damage [40, 41]. Furthermore, the relatively modest sample size of gout patients in our study may have influenced these findings, underscoring the need for larger-scale research to confirm these observations. In summary, UHR exhibits pronounced disease-specificity in risk assessment across various populations, necessitating tailored clinical interpretations based on individual metabolic profiles and comorbidities. Subgroup analyses revealed no significant variations in UHR-mortality associations across sex, BMI, or age groups (all p-interaction >0.05). The consistent link between UHR levels and mortality in gout patients has been highlighted across diverse clinical settings.

One of the key strengths of our research lies in the successful enrollment of a sizable U.S.-based gout cohort, which provided robust and detailed data. We systematically accounted for potential confounders, including demographic factors, comorbidities, and biochemical markers, while leveraging mortality data from extended follow-up periods to bolster the study’s reliability. Nevertheless, we must recognize certain limitations. This study has several limitations. Firstly, the diagnosis of gout is based on self-reporting, which may lead to misclassification bias. Secondly, UHR is only a single baseline measurement value and cannot reflect its dynamic changes. Moreover, although multiple confounding factors have been adjusted, there may still be unmeasured residual confounding (such as the use of uric acid-lowering and lipid-lowering drugs, etc.). NHANES lacks systematic medication data, so the direct influence of drugs on uric acid and HDL-c levels has not been controlled, which may affect the estimation of the association between UHR and mortality risk. However, as UHR is a composite indicator, its value itself can partially reflect the metabolic status after treatment, and relevant clinical indicators have been included in the model, which can alleviate this limitation to a certain extent. Finally, this study is based on the American population, and caution is needed when extrapolating to other populations. Further verification is needed by combining medication records, repeated measurement of UHR, and prospective studies that diagnose gout clinically.

## Conclusion

In conclusion, we found that risk of all-cause mortality and risk of cardiovascular-related death in gout patients are significantly increased with increasing UHR levels. The present study confirms that UHR has significant clinical value in prognostic assessment and mortality risk prediction of gout patients, and its application as a novel biomarker deserves in-depth exploration. Follow-up studies may aim to develop precise intervention strategies based on UHR modulation with a view to optimizing the clinical regression of gout patients.

## Supplementary Information

Supplementary Material 1.

Supplementary Material 2.

Supplementary Material 3.

## Data Availability

No datasets were generated or analysed during the current study.

## References

[1] Choi HK, Mount DB, Reginato AM, American college of Physicians, American physiological Society. Pathogenesis of gout. Ann Intern Med. 2005;143(7):499–516. 16204163 10.7326/0003-4819-143-7-200510040-00009

[2] Zhang W, Doherty M, Pascual E, et al. EULAR evidence based recommendations for gout. Part I: diagnosis. Report of a task force of the standing committee for international clinical studies including therapeutics (ESCISIT). Ann Rheum Dis. 2006;65(10):1301–11. 16707533 10.1136/ard.2006.055251 PMC1798330

[3] Doherty M. New insights into the epidemiology of gout. Rheumatol (Oxf Engl). 2009;48(Suppl 2):ii2–8. 10.1093/rheumatology/kep086 19447779

[4] Feig DI, Kang DH, Johnson RJ. Uric acid and cardiovascular risk. N Engl J Med. 2008;359(17):1811–21. 18946066 10.1056/NEJMra0800885 PMC2684330

[5] Smith EUR, Díaz-Torné C, Perez-Ruiz F, et al. Epidemiology of gout: an update. Best Pract Res Clin Rheumatol. 2010;24(6):811–27. 21665128 10.1016/j.berh.2010.10.004

[6] Brook RD, Yalavarthi S, Myles JD, et al. Determinants of vascular function in patients with chronic gout. J Clin Hypertens Greenwich Conn. 2011;13(3):178–88. 10.1111/j.1751-7176.2010.00406.x PMC3076024 21366849

[7] Aktas G, Khalid A, Kurtkulagi O, et al. Poorly controlled hypertension is associated with elevated serum uric acid to HDL-cholesterol ratio: a cross-sectional cohort study. Postgrad Med. 2022;134(3):297–302. 35142235 10.1080/00325481.2022.2039007

[8] Lim S, Shen et al. Association of variability in uric acid and future clinical outcomes of patients with coronary artery disease undergoing percutaneous coronary intervention. Atherosclerosis. 2020; 297: 40–46. 10.1016/j.atherosclerosis.2020.01.025 32062138

[9] Welty FK. How do elevated triglycerides and low HDL-cholesterol affect inflammation and atherothrombosis? Curr Cardiol Rep. 2013;15(9):400. 23881582 10.1007/s11886-013-0400-4 PMC4465984

[10] Averna M, Stroes E. How to assess and manage cardiovascular risk associated with lipid alterations beyond LDL. Atheroscler Suppl. 2017;26:16–24. 28434480 10.1016/S1567-5688(17)30021-1

[11] Wen S, Arakawa H, Tamai I. Uric acid in health and disease: from physiological functions to pathogenic mechanisms. Pharmacol Ther. 2024;256:108615. 38382882 10.1016/j.pharmthera.2024.108615

[12] Mocciaro G, D’Amore S, Jenkins B, et al. Lipidomic approaches to study HDL metabolism in patients with central obesity diagnosed with metabolic syndrome. Int J Mol Sci. 2022;23(12):6786. 35743227 10.3390/ijms23126786 PMC9223701

[13] Hui N, Barter PJ, Ong KL, et al. Altered HDL metabolism in metabolic disorders: insights into the therapeutic potential of HDL. Clin Sci. 2019;133(21):2221–35. 10.1042/CS20190873 31722013

[14] Yang Y, Shen XY, Tang HX et al. Sex differences in the association of the uric acid to high-density lipoprotein cholesterol ratio with coronary artery disease risk among Chinese nondialysis patients with CKD stages 3–5. Nutr metab cardiovasc dis: NMCD. 2024; 34 (6): 1546–53. 10.1016/j.numecd.2024.03.003 38555242

[15] Aydın C, Emlek N. The relationship between uric acid to high-density lipoprotein cholesterol ratio and collateral index in patients with chronic total occlusion. Kardiologiia. 2021;61(9):61–5. 34713787 10.18087/cardio.2021.9.n1750

[16] Kurtkulagi O, Tel BMA, Kahveci G, et al. Hashimoto’s thyroiditis is associated with elevated serum uric acid to high density lipoprotein-cholesterol ratio. Rom J Intern Med. 2021;59(4):403–8. 34142519 10.2478/rjim-2021-0023

[17] Kosekli MA, Kurtkulagii O, Kahveci G et al. The association between serum uric acid and high density lipoprotein-cholesterol ratio and non-alcoholic fatty liver disease: the abund study. Rev Assoc Med Bras (1992). 2021; 67 (4): 549–554. 10.1590/1806-9282.20201005 34495059

[18] Kocak MZ, Aktas G, Erkus E, et al. Serum uric acid to HDL-cholesterol ratio is a strong predictor of metabolic syndrome in type 2 diabetes mellitus. Rev Assoc Med Bras (1992). 2019;65(1):9–15. 30758414 10.1590/1806-9282.65.1.9

[19] Yazdi F, Baghaei MH, Baniasad A, et al. Investigating the relationship between serum uric acid to high-density lipoprotein ratio and metabolic syndrome. Endocrinology, Diabetes & Metabolism. 2022;5(1):e00311. 10.1002/edm2.311 PMC8754234 34705333

[20] Liu R, Peng Y, Wu H, et al. Uric acid to high-density lipoprotein cholesterol ratio predicts cardiovascular mortality in patients on peritoneal dialysis. Nutr Metab Cardiovasc Dis. 2021;31(2):561–9. 33223397 10.1016/j.numecd.2020.10.005

[21] Kong Y, Lin M, Fu Y, Huang B, Jin M, Ma L. Elevated log uric acid-to-high-density lipoprotein cholesterol ratio (UHR) as a predictor of increased female infertility risk: insights from the NHANES 2013–2020. Lipids Health Dis. 2025;24(1):127. 40170047 10.1186/s12944-025-02521-w PMC11963525

[22] Yi Y, Luo Q, Chen J, et al. Association between the uric acid-to-HDL-cholesterol ratio (UHR) and the risk of cardiovascular disease and dyslipidemia: a population-based study. Lipids Health Dis. 2025;24(1):143. 40241174 10.1186/s12944-025-02551-4 PMC12001538

[23] Yang Y, Zhang J, Jia L, et al. Uric acid to high-density lipoprotein cholesterol ratio predicts adverse cardiovascular events in patients with coronary chronic total occlusion. Nutr Metab Cardiovasc Dis. 2023;33(12):2471–8. 37586923 10.1016/j.numecd.2023.07.037

[24] Li X, Guan B, Wang Y, et al. Association between high-density lipoprotein cholesterol and all-cause mortality in the general population of Northern China. Sci Rep. 2019;9(1):14426. 31594968 10.1038/s41598-019-50924-4 PMC6783426

[25] Bowe B, Xie Y, Xian H, et al. High density lipoprotein cholesterol and the risk of all-cause mortality among U.S. veterans. Clin J Am Soc Nephrol. 2016;11(10):1784–93. 27515591 10.2215/CJN.00730116 PMC5053782

[26] Lamacchia O, Fontana A, Pacilli A, et al. On the non-linear association between serum uric acid levels and all-cause mortality rate in patients with type 2 diabetes mellitus. Atherosclerosis. 2017;260:20–6. 28334637 10.1016/j.atherosclerosis.2017.03.008

[27] Song Z, Deng D, Wu H. Association of serum uric acid to all-cause and cardiovascular mortality in patients with cardiovascular disease. Sci Rep. 2024;14(1):21808. 39294202 10.1038/s41598-024-72527-4 PMC11410977

[28] Pérez Ruiz F, Richette P, Stack AG, et al. Failure to reach uric acid target of < 0.36 mmol/L in hyperuricaemia of gout is associated with elevated total and cardiovascular mortality. RMD Open. 2019;5(2):e001015. 31673414 10.1136/rmdopen-2019-001015 PMC6803010

[29] Sugihara S, Hisatome I, Kuwabara M, et al. Depletion of uric acid due to SLC22A12 (URAT1) loss-of-function mutation causes endothelial dysfunction in hypouricemia. Circ J. 2015;79(5):1125–32. 25739858 10.1253/circj.CJ-14-1267

[30] Park B, Jung DH, Lee YJ. Predictive value of serum uric acid to HDL cholesterol ratio for incident ischemic heart disease in non-diabetic Koreans. Biomedicines. 2022;10(6):1422. 35740443 10.3390/biomedicines10061422 PMC9219787

[31] Xuan Y, Zhang W, Wang Y, et al. Association between uric acid to HDL cholesterol ratio and diabetic complications in men and postmenopausal women. Diabetes Metab Syndr Obes: Targets Ther. 2023;16:167–77. 10.2147/DMSO.S387726 PMC9869791 36760595

[32] Yang Y, Shen X, yan, Tang H et al. xia,. Sex differences in the association of the uric acid to high-density lipoprotein cholesterol ratio with coronary artery disease risk among Chinese nondialysis patients with CKD stages 3–5. Nutr Metab Cardiovas. 2024; 34 (6): 1546–1553. 10.1016/j.numecd.2024.03.003 38555242

[33] Liu R, Peng Y, Wu H, et al. Uric acid to high-density lipoprotein cholesterol ratio predicts cardiovascular mortality in patients on peritoneal dialysis. Nutr Metab Cardiovasc Dis. 2021;31(2):561–9. 33223397 10.1016/j.numecd.2020.10.005

[34] Copur S, Demiray A, Kanbay M. Uric acid in metabolic syndrome: does uric acid have a definitive role? Eur J Intern Med. 2022;103:4–12. 35508444 10.1016/j.ejim.2022.04.022

[35] Kimura Y, Yanagida T, Onda A, Tsukui D, Hosoyamada M, Kono H. Soluble uric acid promotes atherosclerosis via AMPK (AMP-activated protein kinase)-mediated inflammation. Arterioscler Thromb Vasc Biol. 2020;40(3):570–82. 31996020 10.1161/ATVBAHA.119.313224

[36] Beazer JD, Patanapirunhakit P, Gill JMR, et al. High-density lipoprotein’s vascular protective functions in metabolic and cardiovascular disease - could extracellular vesicles be at play? Clin Sci (Lond). 2020;134(22):2977–86. 33210708 10.1042/CS20200892

[37] Ding L, Guo H, Zhang C, et al. Serum uric acid to high-density lipoprotein cholesterol ratio is a predictor for all-cause and cardiovascular disease mortality in patients with diabetes: evidence from NHANES 2005–2018. Nutr Metab Cardiovasc Dis. 2024;34(11):2480–8. 39174432 10.1016/j.numecd.2024.07.001

[38] Chen-Xu M, Yokose C, Rai SK, et al. Contemporary prevalence of gout and hyperuricemia in the united States and decadal trends: the National health and nutrition examination survey, 2007–2016. Arthritis Rheumatol. 2019;71(6):991–9. 30618180 10.1002/art.40807 PMC6536335

[39] Faires JS, McCarty DJ. Acute arthritis in man and dog after intrasynovial injection of sodium urate crystals. Lancet. 1962;280:682–5. 10.1016/s0140-6736(62)91050-4 13979409

[40] Torimoto K, Okada Y, Tanaka Y. [Type 2 diabetes and vascular endothelial Dysfunction]. JUOEH. 2018;40(1):65–75. 10.7888/juoeh.40.65 29553076

[41] Demir S, Nawroth PP, Herzig S, et al. Emerging targets in type 2 diabetes and diabetic complications. Adv Sci. 2021;8(18):e2100275. 10.1002/advs.202100275 PMC8456215 34319011

