# Supplementary material for: Association of serum uric acid to high-density lipoprotein cholesterol ratio with all-cause mortality and cardiovascular disease mortality in patients with gout
Source: BMC Cardiovasc Disord. 2025 Nov 19;25:821. doi: 10.1186/s12872-025-05254-x (PMC12628874; doi:10.1186/s12872-025-05254-x)
Supplement: Supplementary file 1 — Supplementary Material 1. [file 12872_2025_5254_MOESM1_ESM.docx]

| UHR quantiles | Model 4 | | Model 5 | | Model 6 | |
| --- | --- | --- | --- | --- | --- | --- |
|  | HR (95%CI) | P-value | HR (95%CI) | P-value | HR (95%CI) | P-value |
| All-cause mortality | | | | | | |
| UHR (per 10% increment) | 1.25 (1.05, 1.50) | 0.014 | 1.25 (1.05, 1.48) | 0.010 | 1.19 (1.01, 1.40) | 0.042 |
| UHR quartile |  |  |  |  | | |
| Q1 | 1.23 (0.90, 1.69) | 0.200 | 1.14 (0.86, 1.52) | 0.356 | 1.26 (0.93, 1.71) | 0.138 |
| Q2 | Reference |  | Reference |  | Reference |  |
| Q3 | 1.41 (1.03, 1.93) | 0.030 | 1.22 (0.92, 1.62) | 0.166 | 1.35 (0.99, 1.85) | 0.060 |
| Q4 | 1.35 (0.98, 1.87) | 0.070 | 1.30 (0.97, 1.74) | 0.083 | 1.24 (0.90, 1.69) | 0.188 |
| P for trend |  | 0.040 |  | 0.073 |  | 0.147 |
| Cardiovascular mortality | | | | | | |
| UHR (per 10% increment) | 1.40 (1.04, 1.90) | 0.026 | 1.44 (1.10, 1.90) | 0.008 | 1.23 (0.94, 1.62) | 0.136 |
| UHR quartile |  |  |  |  | | |
| Q1 | 1.10 (0.65, 1.86) | 0.728 | 0.96 (0.59, 1.56) | 0.872 | 1.16 (0.69, 1.94) | 0.578 |
| Q2 | Reference |  | Reference |  | Reference |  |
| Q3 | 1.59 (0.96, 2.63) | 0.073 | 1.39 (0.88, 2.20) | 0.157 | 1.58 (0.95, 2.62) | 0.076 |
| Q4 | 1.34 (0.77, 2.32) | 0.305 | 1.39 (0.85, 2.27) | 0.189 | 1.11 (0.64, 1.93) | 0.697 |
| P for trend |  | 0.148 |  | 0.111 |  | 0.404 |

Table S1

Model 4: Removed three extreme UHR values (total sample size 1,476), adjusted for age, gender, race, DBP, SBP, WBC count, RBC count, platelet count, neutrophil count, Cre, ALT, LDH, TG, K+, Cl-, Na+, TP, heart attack, stroke, cancer, and diabetes.

Model 5: Included 177 participants with missing UHR data, with sensitivity analysis performed using random forest imputation (total sample size 1,656). Adjusted for age, gender, race, DBP, SBP, WBC count, RBC count, platelet count, neutrophil count, Cre, ALT, LDH, TG, K+, Cl-, Na+, TP, heart attack, stroke, cancer, and diabetes.

Model 6: Sensitivity analysis conducted by re-screening covariates using LASSO regression. Adjusted for age, RBC count, Cre, LDH, neutrophil count, BMI, and stroke.
