# Supplementary material for: Association of serum uric acid to high-density lipoprotein cholesterol ratio with all-cause mortality and cardiovascular disease mortality in patients with gout
Source: BMC Cardiovasc Disord. 2025 Nov 19;25:821. doi: 10.1186/s12872-025-05254-x (PMC12628874; doi:10.1186/s12872-025-05254-x)
Supplement: Supplementary file 2 — Supplementary Material 2. [file 12872_2025_5254_MOESM2_ESM.docx]

| Characteristic | UHR | | P-value |
| --- | --- | --- | --- |
|  | Participants lacking UHR | Included participants |  |
| N of participants | 117 | 1479 |  |
| Age (years) | 65.94 ± 14.32 | 63.96 ± 12.98 | 0.058 |
| SBP (mmHg) | 138.44 ± 23.42 | 132.97 ± 21.01 | 0.011 |
| DBP (mmHg) | 70.44 ± 15.21 | 70.14 ± 15.16 | 0.848 |
| WBC count (10^9^/l) | 7.97 ± 2.51 | 7.54 ± 3.20 | 0.404 |
| RBC count (10^12^/l) | 4.55 ± 0.52 | 4.61 ± 0.58 | 0.552 |
| Platelet count (10^9^/l) | 229.08 ± 70.62 | 227.96 ± 67.42 | 0.918 |
| Cre (mg/dL) | - | 1.03 (0.87-1.28) | - |
| ALT (U/L) | - | 21.00 (16.00-29.00) | - |
| TG (mg/dL) | - | 7.13 ±0.52 | - |
| LDH (U/L) | - | 144.31 ± 38.76 | - |
| Na + (mmol/L) | - | 139.49 ± 2.76 | - |
| K + (mmol/L) | - | 4.09 ± 0.42 | - |
| Cl^-^ (mmol/L) | - | 102.74 ± 3.68 | - |
| TP (g/L) | - | 7.13 ± 0.52 | - |
| Neutrophils count (10^9^/l) | 4.97 ± 1.91 | 4.51 ± 1.77 | 0.109 |
| BMI (kg/m^2^) | 32.63 ± 8.36 | 31.72 ± 7.42 | 0.218 |
| Sex (%) |  |  | 0.008 |
| Male | 59.89% | 69.64% |  |
| Female | 40.11% | 30.36% |  |
| Race (%) |  |  | <0.001 |
| Mexica American | 6.21% | 7.17% |  |
| Other Hispanic | 3.95% | 5.95% |  |
| Non-Hispanic White | 34.46% | 49.49% |  |
| Non-Hispanic Black | 45.20% | 25.56% |  |
| Others | 10.17% | 11.83% |  |
| Heart attack (%) |  |  | 0.910 |
| Yes | 14.69% | 15.01% |  |
| No | 85.31% | 84.99% |  |
| Stroke (%) |  |  | 0.845 |
| Yes | 10.73% | 11.22% |  |
| No | 89.27% | 88.78% |  |
| Cancer (%) |  |  | 0.919 |
| Yes | 20.34% | 20.01% |  |
| No | 79.66% | 79.99% |  |
| Diabetes |  |  | 0.020 |
| Yes | 40.68% | 31.98% |  |
| No | 59.32% | 68.02% |  |

Table S2
