# Supplementary material for: Association of serum uric acid to high-density lipoprotein cholesterol ratio with all-cause mortality and cardiovascular disease mortality in patients with gout
Source: BMC Cardiovasc Disord. 2025 Nov 19;25:821. doi: 10.1186/s12872-025-05254-x (PMC12628874; doi:10.1186/s12872-025-05254-x)
Supplement: Supplementary file 3 — Supplementary Material 3. [file 12872_2025_5254_MOESM3_ESM.docx]

| UHR (%) quartiles | All-cause mortality | |  | Cardiovascular mortality | |  |
| --- | --- | --- | --- | --- | --- | --- |
|  | Events, n/N | Incidence rate per 1000 person, years (95% CI) | | Events, n/N | Incidence rate per 1000 person, years (95% CI) | |
| 3% ≤ Q1 < 10% | 94/370 | 10.8(8.8-13.2) | | 30/370 | 3.5(2.5-5.0) | |
| 10% ≤ Q2 < 14% | 86/369 | 9.9(8.0-12.2) | | 32/369 | 3.7(2.6-5.2) | |
| 14% ≤ Q3 < 19% | 88/369 | 10.1(8.2-12.4) | | 35/369 | 4.0(2.9-5.6) | |
| 19% ≤ Q4 < 78% | 90/371 | 10.4(8.5-12.8) | | 30/371 | 3.5(2.5-5.0) | |

Table S3
